# Reference equations of oxygen uptake for the step test in the obese population

**DOI:** 10.1590/1414-431X2022e11864

**Published:** 2022-03-21

**Authors:** C.F. Fagundes, L. Di Thommazo-Luporini, C.L. Goulart, D. Braatz, A. Ditomaso, A. Borghi-Silva

**Affiliations:** 1 Departamento de Fisioterapia, Universidade Federal de São Carlos, São Carlos, SP, Brasil; 2 Departamento de Engenharia de Produção, Universidade Federal de São Carlos, São Carlos, SP, Brasil; 3 Instituto Federal de Educação, Ciência e Tecnologia de São Paulo, São Carlos, SP, Brasil

**Keywords:** Obesity, Cardiorespiratory fitness, Exercise test, Step test, Oxygen uptake

## Abstract

The aim of this study was to establish reference equations for the six-minute step test (6MST) based on demographic, anthropometric, body composition, and performance variables able to predict oxygen uptake (V̇O_2_) in obese individuals. Seventy-three obese adults (42±14 years old, body mass index >30 kg/m^2^) from both sexes were included. They underwent anamnesis, body composition evaluation, and the 6MST with expired gases registered simultaneously. Three equations were developed for the obese population (n=73; 59% female). The first equation was composed of the up-and-down step cycles (UDS), sex, and age as predictors; the second equation was composed of the UDS, age, and lean mass (LM). Both equations collectively explained 68.1% of the V̇O_2_ variance in the 6MST, while the third equation, composed of the UDS, age, and body mass, accounted for 67.7% of the V̇O_2_ variance. UDS, sex, age, LM, and body mass were important V̇O_2_ predictors of 6MST in these obese individuals. This study contributes to the dissemination of a simple, inexpensive, and fast evaluation method that can provide important indicators of cardiorespiratory fitness and guide strategies for rehabilitation.

## Introduction

Obesity is considered a non-communicable chronic disease with alarming global proportions (1). It leads to several adverse pulmonary, cardiac, and metabolic effects (2,3). In addition to the mechanical disadvantage of excessive weight, obese individuals have a significant functional limitation of muscular performance, affecting activities of daily living (4,5). In this context, several authors have studied the deleterious effects of obesity on cardiorespiratory fitness (CRF) and functional capacity (6-9), focusing on physical rehabilitation as one of the strategies for treatment.

Oxygen uptake (V̇O_2_) is the gold standard parameter to quantify CRF and it reflects the individual's ability to capture, transport, and metabolize oxygen during muscle contraction (10). However, V̇O_2_ is usually obtained by means of the maximal or symptom-limited cardiopulmonary exercise test (CPX) (11,12), which is used only in large health centers or research institutions due to its high complexity, the cost of the equipment, and the need for trained staff (13).

Thus, functional exercise tests have been the preferred option in clinical practice to evaluate individuals with health limitations (8,14-20), since they are simpler and cheaper than the CPX and elicit submaximal or near maximal effort. Additionally, they can predict maximal performance and/or V̇O_2_ in healthy and disabled populations from different functional exercises, such as walking or stepping tests (6,18,21-23).

The step test has been largely used in cardiometabolic and perceptual assessments (14), and it can be used where physical space is restricted. However, the literature is still scarce with respect to V̇O_2_ prediction equations based on the step test performance in obese individuals of both sexes and broad age groups. Because the step test is a viable, inexpensive, and easy to perform field test, the possibility of having V̇O_2_ prediction equations would enable individualized physical rehabilitation for the obese population.

Thus, the first aim of this study was to establish V̇O_2_ reference equations for the six-minute step test (6MST) for the obese population in the age range from 20 to 79 years, based on demographic, anthropometric, body composition, and performance variables. The second aim was to verify if there would be a difference according to sex in predictors of V̇O_2_ in the 6MST. The hypothesis of the study is that a V̇O_2_ prediction equation can be derived from performance in the 6MST in the obese population and that different predictors will be included in the reference equations according to sex.

## Material and Methods

### Design

This cross-sectional study was conducted in the cardiopulmonary physiotherapy laboratory of Universidade Federal de São Carlos (Brazil). The total sample was recruited from the community via social communication within the department and university as well as social media advertisements from January 2017 to March 2018.

### Subjects

The inclusion criteria were individuals of both sexes aged 20 to 79 years, with a clinical diagnosis of obesity (BMI ≥30 kg/m^2^) and a sedentary lifestyle (i.e., no more than 150 min per week of moderate physical activity) (24). Individuals who had a diagnosis of chronic obstructive pulmonary disease and other respiratory, cardiovascular, or metabolic diseases that could elicit or exacerbate dyspnea during effort, pregnancy, current smokers, people with diabetes mellitus, serious cognitive impairment proven by the Mini-Mental State Examination (MMSE) if older than 60 years old (25), and any contraindication or condition that could compromise the performance of the 6MST were not eligible for the study. Exclusion criteria were inadequate arterial blood pressure response to exercise, forced expiratory volume in one second (FEV_1_) <70% on spirometry, or refusal to participate in the study. After agreeing to participate in the study, all included volunteers signed an informed consent form approved by the Human Ethics Committee of the University (CAAE: 68132517.2.0000.5504; decision number: 2.226.612).

### Experimental procedures

Participants were submitted to 2 days of evaluation. On the first day, anamnesis, Baecke physical activity questionnaire, and physical examination were performed. Weight and body composition variables (percent body fat [BF%] and lean mass [LM]) were collected with the tetrapolar bioelectrical impedance InBody 720 device (Biospace, South Korea) according to the manufacturer's guidelines. All individuals were advised to wear light clothing, to present 4 h after absolute fasting, and to urinate prior to the assessment. Women of reproductive age were evaluated at the follicular phase of their menstrual cycle to avoid a bias from hormonal effects. The follicular phase of the volunteers' cycle was taken from the self-reported profile in the anamnesis. Assessments were performed between the first and the day before ovulation (usually until the 13th day in women with a regular 28-day menstrual cycle). In women with irregular menstrual cycles, assessments were performed within the first 10 days of the menstrual cycle. The individuals also completed at least three acceptable maximal forced and slow expiratory maneuvers according to standardized procedures to complete the pulmonary function assessment (26). A portable ergospirometry system (Oxycon Mobile^®^, Germany) was used after adequate calibration.

### Six-minute step test

On the second day, the 6MST was applied. Participants were instructed to go up and down on a 15-cm-high step ergometer in free cadence as fast as possible, starting the movement with the dominant leg. The step ergometer was previously developed and registered in the National Institute of Industrial Property (INPI, registration number #BR 20 2015 000603 4), the official government agency for industrial property rights in Brazil. This equipment represents an economically viable technology, since it is coupled to software and is easy to use and interpret, taking up little space in the physical fitness evaluation.

During the exercise test, subjects were allowed to slow down or increase the pace and even stop if deemed necessary, according to symptoms of dyspnea and/or fatigue. The CR-10 Borg scale (27) was applied at the peak of exercise to monitor the perceived exertion. Standardized encouraging phrases according to the American Thoracic Society guidelines for the six-minute walk test (28) were given to the volunteers as well as the remaining test time. The UDS was recorded every minute, and the total number completed during the 6MST was used as a performance measure. Heart rate (HR) was recorded with a cardio-frequency meter, the Polar S810i telemetry system (Polar^®^, Finland), and arterial blood pressure was measured with a standard cuff sphygmomanometer (Diasyst^®^, Brazil) at rest (sitting) and at the peak of the 6MST.

### Ventilatory, metabolic, cardiac, and hemodynamic measurements

Expired gases were collected during the 6MST with the portable Oxycon Mobile^®^ to analyze V̇O_2_, carbon dioxide production (V̇CO_2_), respiratory exchange rate (RER), minute ventilation (V̇_E_), and breathing frequency (BF). All metabolic and ventilatory data were processed in mobile averages every 15 s, and the peak variables were defined as the average of the highest values obtained at the end of the six minutes. This observational study was conducted according to the STROBE standards (29).

### Statistical analysis

A sample size of 55 individuals was estimated *a priori* by the GPower statistical program, version 3.1.3 (Franz Faul Universität Kiel, Germany) based on the multiple linear regression model, considering a 5% type I error, 95% power, and 0.25 effect size, as well as eight potential predictors to be included in the equation (up-and-down step cycles [UDS], sex, age, body mass, stature, body mass index [BMI], BF%, and LM in kg).

Data were analyzed using the SPSS Statistics for Windows, Version 20.0 (SPSS Inc., USA) software package. Data distribution was verified by the Kolmogorov-Smirnov test. Data are reported as means and 95% confidence intervals. For further elucidation, the sample was characterized as total population (n=73) and for women (n=43) and men (n=30).

One-way ANOVA with *post hoc* Tukey test or Kruskal-Wallis test with *post hoc* Mann-Whitney test (for weight, LM, BMI, forced vital capacity [FVC], F_EV1_, F_EV1_/FVC, resting HR, systolic blood pressure [SBP], diastolic blood pressure [DBP], Baecke's score, dyspnea, lower limb fatigue, V̇_E_, and RER) were used depending on parametric and nonparametric assumptions.

The stepwise multiple linear regression model was used to predict V̇O_2_ at the peak of 6MST considering the following potential predictors: UDS, sex, age, body mass, stature, BMI, BF%, and LM for the total population. Statistical significance was set at P<0.05 or <0.02 (Bonferroni adjustment for multiple comparisons).

## Results

**Figure 1:**
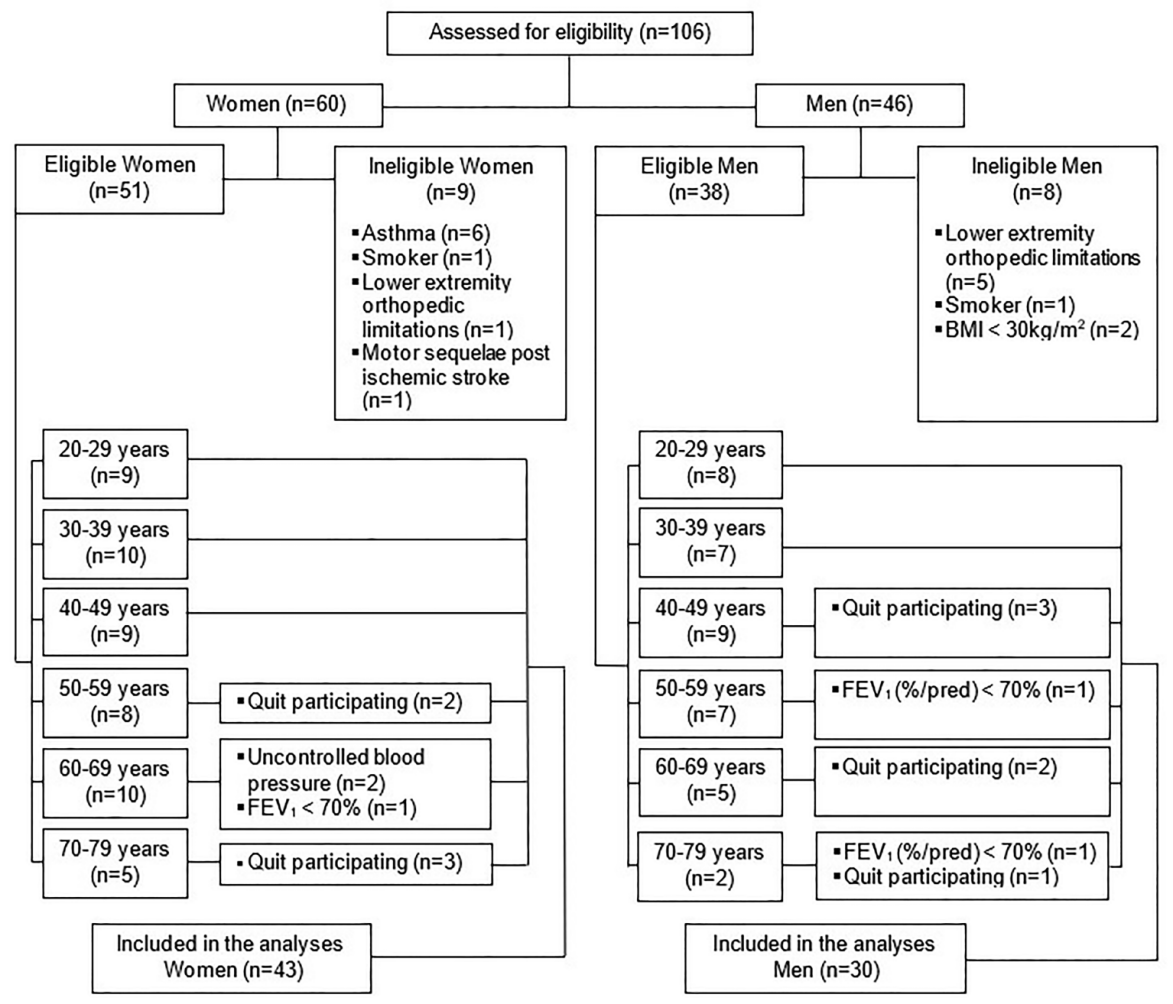
Volunteer recruitment flowchart including sample loss.

The flow diagram in Figure 1 shows the recruitment, eligible individual assessment, number and causes of sample loss, and the sample included in the final analyses. Of the 106 potential study participants assessed for eligibility, almost 69% of them were included. Of these, 58.9% were women. We displayed the number of volunteers in each decade of life. The characteristics of volunteers are reported in Table 1. All the volunteers had normal pulmonary function, and the total sample was classified as obese grade II based on the mean BMI (38.5±5.4 kg/m^2^). As indicated by Baecke's questionnaire, our sample was classified as sedentary.

**Table 1:** Demographic, anthropometric, body composition, physical activity level, and lung function of the volunteers.

	Total sample (n=73)	Women (n=43)	Men (n=30)
Age (years)	42 (39.4 to 45.9)	43 (38.9 to 48.2)	41 (36.6 to 46.1)
Height (m)	1.66 (1.64 to 1.68)	1.60 (1.58 to 1.62)*	1.74 (1.72 to 1.76)^†‡^
Weight (kg)	106.7 (101.0 to 111.5)	98.4 (93.7 to 103.2)	118.6 (110.7 to 126.6)^†‡^
BF%	42.3 (40.6 to 44.1)	46.9 (45.6 to 48.3)*	35.6 (33.4 to 37.9)^†‡^
LM (kg)	58.4 (55.0 to 61.8)	49.0 (47.1 to 50.9)*	71.8 (67.2 to 76.5)^†‡^
BMI (kg/m^2^)	38.4 (37.2 to 39.7)	38.0 (36.6 to 39.6)	39.0 (37.8 to 41.4)
Baecke questionnaire (total mean)	2.2 (2.1 to 2.3)	2.3 (2.2 to 2.4)	2.0 (1.9 to 2.2)^‡^
FVC (% pred)	95.2 (91.3 to 99.1)	99.7 (94.0 to 105.4)	88.7 (84.5 to 93.1)^‡^
F_EV1_ (% pred)	94.4 (91.2 to 97.7)	99.2 (95.0 to 103.7)	87.4 (83.6 to 91.3)^‡^
F_EV1_/FVC (% pred)	99.7 (98.0 to 101.5)	100.48 (97.8 to 103.1)	98.78 (96.7 to 100.9)

Data are reported as means and 95% confidence intervals. BF%: percentage of body fat; LM: lean mass; BMI: body mass index; FVC: forced vital capacity; F_EV1_: forced expiratory volume in one second. *^†^P<0.05 *vs* total sample; ^‡^P<0.05 *vs* women (one-way ANOVA with *post hoc* Tukey test or Kruskal-Wallis test with *post hoc* Mann-Whitney test).

The sample of obese men had higher values of height, weight, and LM as well as lower values of BF% than the total sample and obese women. Additionally, lower levels of physical activity (Baecke's questionnaire), FVC, and F_EV1_ were found in obese men compared to obese women. Women had lower height and LM as well as higher BF% compared to the total sample.

Table 2 displays the metabolic, ventilatory, cardiovascular, and performance variables at rest and at the peak of 6MST. Obese women had lower values for resting HR, peak SBP, V̇O_2_ (in mL/min and %pred), V̇CO_2_, and V̇_E_ as well as higher values of perceived dyspnea compared to men and lower peak SBP and higher V̇O_2_ (%pred) compared to the total sample. Obese men had higher peak SBP and V̇_E_ as well as lower V̇O_2_ (%pred) compared to the total sample.

**Table 2:** Metabolic, ventilatory, and performance variables at rest and at the peak of the 6MST.

Physiological variables	Total (n=73)	Women (n=43)	Men (n=30)
Rest			
HR (bpm)	82 (79 to 85)	78 (75 to 80)	86 (82 to 93)^‡^
SBP (mmHg)	128 (127 to 131)	128 (124 to 132)	129 (125 to 134)
DBP (mmHg)	86 (84 to 89)	87 (83 to 90)	86 (83 to 89)
Peak of 6MST			
HR (bpm)	145 (140 to 151)	143 (135 to 150)	149 (141 to 157)
HR (%pred)	82 (79 to 84)	81 (79 to 85)	83 (78 to 86)
SBP (mmHg)	156 (146 to 166)	133 (124 to 141)*	190 (174 to 205)^†‡^
DBP (mmHg)	91 (85 to 96)	87 (81 to 92)	96 (86 to 106)
Dyspnea (0-10)	3 [0.5-10]	4 [0.5-10]	3 [0-7]^‡^
Fatigue (0-10)	3 [0-10]	3 [0-10]	3 [0.5-8]
Metabolic and ventilatory data			
V̇O_2_ (mL·min^-1^)	1718 (1618 to 1816)	1607 (1483 to 1731)	1874 (1724 to 2023)^‡^
V̇O_2_ (mL·kg^-1^·min^-1^)	16.3 (15 to 17)	16.4 (15 to 18)	16.1 (15 to 17)
V̇O_2_ (%pred)	78 (74 to 82)	86 (72 to 83)*	66 (71 to 84)^†‡^
V̇O_2_ (mL·min^-1^)	1786 (1672 to 1901)	1633 (1502 to 1766)	1999 (1814 to 2185)^‡^
RER	1.03 (1.02 to 1.06)	1.02 (1.00 to 1.04)	1.06 (1.03 to 1.09)
V̇_E_ (L·min^-1^)	59 (54 to 63)	51 (47 to 55)	69 (61 to 77)^†‡^
BF (per min)	33 (32 to 35)	33 (31 to 35)	37 (31 to 38)
Performance			
UDS	146 (140 to 152)	142 (134 to 152)	151 (143 to 159)

Data are reported as means and 95% confidence interval. Dyspnea and fatigue are reported as number and range. 6MST: six-minute step test; HR: heart rate; SBP: systolic blood pressure; DBP: diastolic blood pressure; V̇O_2_: oxygen uptake; V̇CO_2_: carbon dioxide production; RER: respiratory exchange ratio; V̇_E_: minute ventilation; BF: breathing frequency; UDS: up-and-down step cycle. *^†^P<0.05 compared to total sample; ^‡^P<0.05 compared to women (one-way ANOVA with *post hoc* Tukey test or Kruskal-Wallis test with *post hoc* Mann-Whitney test).

There were two missing values for body composition (BF% and LM) and one each for the ergospirometric variables (V̇O_2_, V̇CO_2_, V̇_E_, BF, and RER) at the peak of 6MST that were treated as missing data and not replaced with an average value for the final analysis.

Considering the total sample (n=73), the univariate analysis demonstrated that V̇O_2_ during 6MST correlated significantly with the UDS (r=0.79; P<0.001) and age (r=-0.55; P<0.001). For women (n=43), the V̇O_2_ during 6MST was significantly correlated with the UDS (r=0.84; P<0.001) and age (r=-0.72; P<0.001). Finally, for the men (n=30), the V̇O_2_ during 6MST was significantly correlated with the UDS (r=0.72; P<0.001).

In the stepwise multiple linear regression analysis for the total sample (n=73), the parameters UDS, sex, age, weight, and LM were able to predict the V̇O_2_ at the peak of 6MST in the three developed equations. These predictors explained 67 to 68% of total variance in relative V̇O_2_ (Table 3). In addition, two other equations were proposed specifically for women in which the predictors taken together (UDS, age, and LM) explained 74 and 75% of the total variance in relative V̇O_2._ One predictive equation was developed for obese men, and the UDS explained 52% of the total variance in V̇O_2_ at the peak of the functional test.

**Table 3:** Reference equations of oxygen uptake in the six-minute step test for obese people.

Groups	Equations
Total sample	V̇O_2_=3.110 + 0.107 × (UDS) - 1.354 × (sex_men=0; women=1_) - 0.049 × (age _years_); r^2^=0.68
	Standard error of the estimate=2.36 mL·kg^-1^·min^-1^
	V̇O_2_=6.549 + 0.103 × (UDS) - 0.065 × (age_years_) - 0.048 × (LM _kg_); r^2^=0.68
	Standard error of the estimate=2.70 mL·kg^-1^·min^-1^
	V̇O_2_=8.252 + 0.096 × (UDS) - 0.067 × (age_years_) - 0.032 × (weight _kg_); r^2^=0.67
	Standard error of the estimate=3.19 mL·kg^-1^·min^-1^
Women	V̇O_2_=5.623 + 0.100 × (UDS) - 0.085 × (age_years_); r^2^=0.75
	Standard error of the estimate=3.44 mL·kg^-1^·min^-1^
	V̇O_2_=-8.422 + 0.129 × (UDS) + 0.127 × (LM _kg_); r^2^=0.74
	Standard error of the estimate=3.26 mL·kg^-1^·min^-1^
Men	V̇O_2_=1.561 + 0.095 × (UDS); r^2^=0.52
	Standard error of the estimate=2.60 mL·kg^-1^·min^-1^

V̇O_2_: oxygen uptake; UDS: up-and-down step cycles; LM: lean mass.

## Discussion

To the best of our knowledge, this is the first study to propose reference equations for V̇O_2_ reached in the free-cadence 6MST for the obese population aged 20 to 79 years and with strong predictors based on performance, anthropometric, and body composition variables. The main findings of our study were: 1) 6MST performance (UDS), sex, age, LM, and body mass were included in V̇O_2_ predictive equations that explained 67 to 68% of V̇O_2_ variance in the 6MST in the three proposed equations for the obese population; 2) UDS, age, and LM were included as predictors in two predictive equations for obese women that explained 74 to 75% of V̇O_2_ variance in the 6MST; and 3) UDS alone explained 52% of the V̇O_2_ variance in the 6MST for obese men.

The evaluation of functional capacity is imperative to set goals for rehabilitation programs and develop weight loss strategies that improve muscular performance, work capacity, and activities of daily living in obese individuals. Predicting the performance of an individual in functional tests is becoming an important evaluation strategy, since it is less expensive, more accessible, and easier to use and interpret compared with the gold standard method for assessing CRF, the CPX, to obtain maximal V̇O_2_. Our findings demonstrated the advantage of the present gold standard measure for assessing CRF level as the main outcome from the predictive equations developed from a submaximal exercise test, the 6MST.

The performance outcome (UDS) had the greatest influence on the V̇O_2_ reached in the 6MST in all the proposed equations. This important parameter directly reflects the individual's performance in a dynamic time-limited exercise involving vertical and horizontal displacement. The strong correlation between USD and V̇O_2_ and the influence of this variable on the prediction of the V̇O_2_ reached at the peak of 6MST corroborated with other findings from our laboratory used to developed predictive equations for estimating V̇O2 at the CPX in obese women from the 6MST (6,14). In the study developed by Carvalho et al. (6), with sedentary obese and lean young women, the UDS accounted for 80% of the correlation with V̇O_2_ at the 6MST peak. This parameter (UDS) was the only predictor selected by the stepwise regression model and accounted for 31% of the total variance of the V̇O_2_ in the same functional test applied in young adult obese women (14).

Differently from both of the above cited studies, our sample included obese people with a large age range from both sexes, and the predictive equations developed were based on the V̇O_2_ reached in the functional exercise test (6MST) and not in the V̇O_2_ expressed at the CPX peak. In this sense, the present study offered the possibility of predicting the V̇O_2_ reached in the 6MST based on parameters that are easy to determine (age, sex, weight, LM, and UDS) allowing researchers to design a more accurate exercise training program for the rehabilitation of obese individuals based on submaximal exercise intensity.

In clinical practice, validated exercise tests to evaluate functional capacity, such as step and walk tests, are widely used in healthy (17,21,30,31) and disabled people (8,15,18,32-34). Arcuri et al. (30) determined the validity of the 6MST and established reference equations based on the 6MST performance using a sample of 91 subjects (42 men and 49 women) with a mean age of 39 years. In contrast, our study included only obese participants, and the proposed reference equations were based on the V̇O_2_ achieved in the 6MST and not on test performance.

The RER is an important indicator of exercise effort, with values above 1.0 reflecting intense effort of the individual (35). In our sample, the RER was 1.02, 1.03, and 1.06 at the peak of the 6MST for the total obese population, women, and men, respectively. This suggests that this functional test required near-maximal effort, at which individuals generally exceeded the anaerobic threshold, which is representative of exercise tolerance and a reliable limit for prescribing physical exercise (36,37). Therefore, RER together with V̇O_2_ demonstrate that 6MST requires efforts between 85-90% of the maximum (14), indicating that this field test can be a useful tool for prescribing submaximal exercise for obese people.

These predictive equations for V̇O_2_ in the 6MST can be an important tool for functional capacity assessment for the obese population, since the CRF has been reported to be a clinical vital sign by the American Heart Association (38). In addition to their applicability in private health clinics and gyms, the advantages of the test requiring less space than walking tests and being inexpensive could also be of interest for future use in the Family Health Program, Basic Health Units, and even Community Centers, which are the gateway to the public health system of the Brazilian population. Thus, the use of the proposed predictive equations can be the basis for the elaboration of effective and individualized exercise rehabilitation protocols for obese people. All equations presented in the present study generally demonstrated good predictive power, but some involved parameters that may be less accessible, such as the measurement of lean mass by bioimpedance. Therefore, it is believed that future studies considering the respective predictive power (R) may confirm the applicability of this important field test using these predictive equations.

### Study limitations

Some limitations of this study must be addressed. Our sample only had two volunteers over the age of 70 years, because this stratum of the population presented more exclusion criteria for participation in the study (presence of chronic respiratory, cardiovascular and/or metabolic diseases, cognitive impairment, and physical limitations to ambulation). Moreover, these individuals had more barriers to participation in the study (e.g., high caregiver dependence and transportation barriers to attend the evaluations). Probably, men’s VO_2_ was lower because they reached a lower percentage of the predicted maximum, especially because the test is submaximal. The small sample affected the predicted values, especially for men. In the specific equation for men, the age factor was not included due to the small sample size. Finally, our sample of elderly people was impaired due to strict health inclusion criteria. Future studies should therefore focus specifically on this population.

### Conclusion

Equations including demographic, anthropometric, body composition, and performance variables were able to predict the V̇O_2_ of 6MST in a sedentary obese population, and specific predictors were selected for reference equations according to sex. The findings of this study contribute to the dissemination of a simple and inexpensive functional test (USD with 15-cm high ergometer in free cadence) that can provide important CRF parameters useful for exercise prescription in primary care and/or rehabilitation of obese people.
